# Hantavirus pulmonary syndrome outbreaks associated with climate variability in Northwestern Argentina, 1997–2017

**DOI:** 10.1371/journal.pntd.0008786

**Published:** 2020-11-30

**Authors:** Ignacio Ferro, Carla M. Bellomo, Walter López, Rocío Coelho, Daniel Alonso, Agostina Bruno, Francisco E. Córdoba, Valeria P. Martinez

**Affiliations:** 1 Instituto de Ecorregiones Andinas—Consejo Nacional de Investigaciones Científicas y Técnicas (CONICET)—Universidad Nacional de Jujuy (UNJu), San Salvador de Jujuy, Argentina; 2 Instituto Nacional de Enfermedades Infecciosas (INEI), Administración Nacional de Laboratorios e Institutos de Salud (ANLIS) “Dr. C. G. Malbrán”, Buenos Aires, Argentina; 3 Instituto de Investigaciones de Enfermedades Tropicales, Oran, Salta, Argentina; 4 Hospital San Vicente de Paul Oran, Salta, Argentina; NIAID Integrated Research Facility, UNITED STATES

## Abstract

**Background:**

Rodent-borne hantaviruses (genus *Orthohantavirus*) are the etiologic agents causing two human diseases: hemorrhagic fever with renal syndrome (HFRS) in Euroasia; and hantavirus pulmonary syndrome (HPS) in North and South America. In South America fatality rates of HPS can reach up to 35%–50%. The transmission of pathogenic hantaviruses to humans occurs mainly via inhalation of aerosolized excreta from infected rodents. Thus, the epidemiology of HPS is necessarily linked to the ecology of their rodent hosts and the contact with a human, which in turn may be influenced by climatic variability. Here we examined the relationship between climatic variables and hantavirus transmission aim to develop an early warning system of potential hantavirus outbreaks based on ecologically relevant climatic factors.

**Methodology and main findings:**

We compiled reported HPS cases in northwestern Argentina during the 1997–2017 period and divided our data into biannual, quarterly, and bimestrial time periods to allow annual and shorter time delays to be observed. To evaluate the relationship of hantavirus transmission with mean temperature and precipitation we used dynamic regression analysis. We found a significant association between HPS incidence and lagged rainfall and temperature with a delay of 2 to 6 months. For the biannual and quarterly models, hantavirus transmission was positively associated with lagged rainfall and temperature; whereas the bimestrial models indicate a direct relationship with the rainfall but inverse for temperature in the second lagged period.

**Conclusions/Significance:**

This work demonstrates that climate variability plays a significant role in the transmission of hantavirus in northwestern Argentina. The model developed in this study provides a basis for the forecast of potential HPS outbreaks based on climatic parameters. Our findings are valuable for the development of public health policies and prevention strategies to mitigate possible outbreaks. Nonetheless, a surveillance program on rodent population dynamics would lead to a more accurate forecast of HPS outbreaks.

## Author summary

Hantavirus pulmonary syndrome (HPS) is a Pan-American emerging disease with a high mortality rate caused by a rodent-borne virus. In Argentina, almost half of the HPS infections occur in the northwestern endemic region. Most of the reported cases (75%) developed severe respiratory insufficiency, of which 30% required mechanical ventilation and 15% with a fatal outcome. In this study area, nearly half of the population is below the poverty line, particularly in rural areas, where most infections occur. Since there are no vaccines currently available nor specific therapeutic treatments, prevention of hantavirus infection involves mainly environmental management practices and educational campaigns. Our results provide a framework for the planning and implementation of early public health prevention campaigns based on the significant relationship between hantavirus outbreaks and delayed climatic variables.

## Introduction

**Fig 1:**
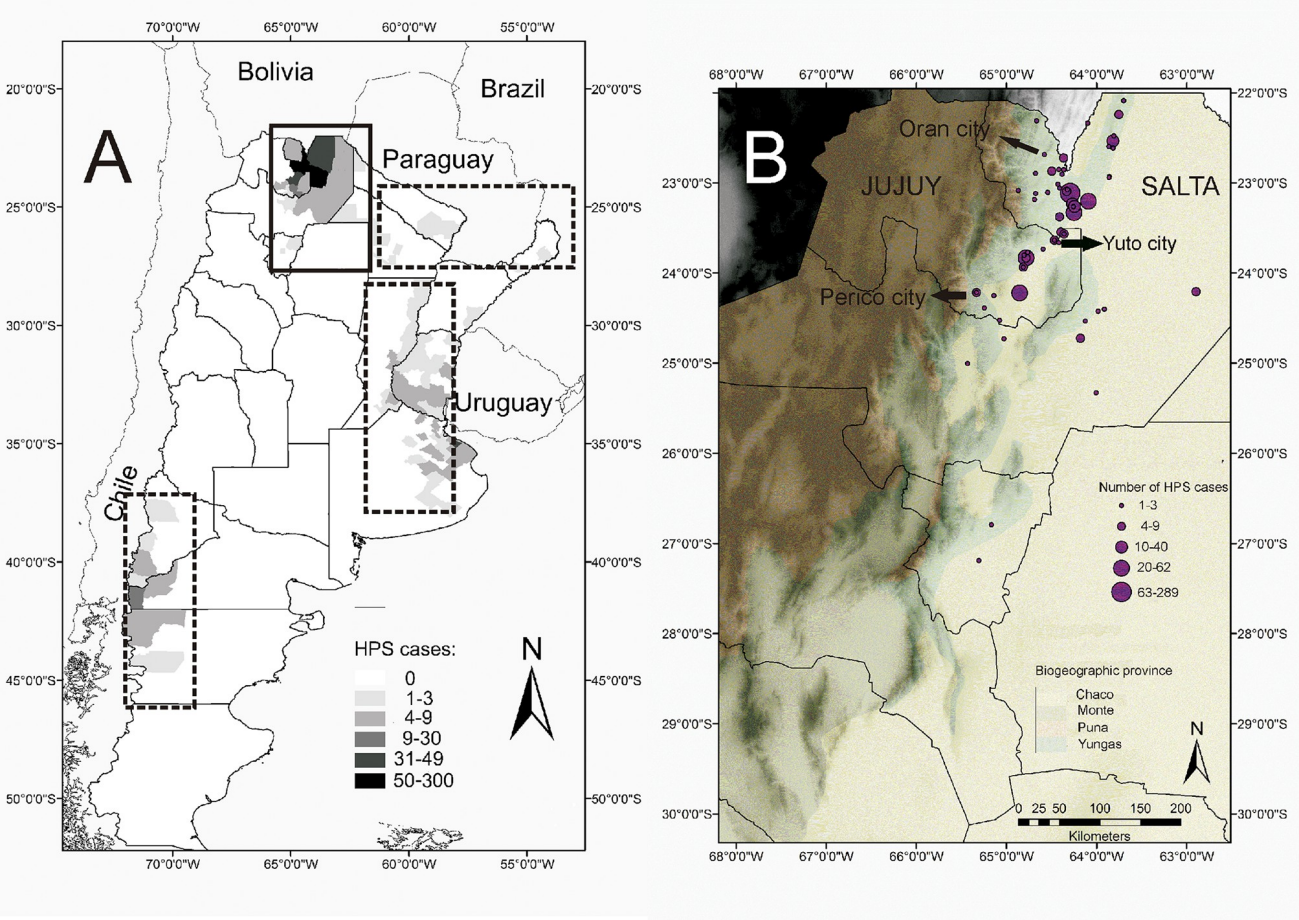
Distribution of hantavirus pulmonary syndrome (HPS). (A) The cumulative number of HPS cases in the 4 endemic regions of Argentina indicated by rectangles and grayscale for every second-level administrative division. (B) Hantavirus pulmonary syndrome cases in Northwestern Argentina; the circles indicate the location of HPS cases and the size of each circle is proportional to the number of cases. Localities are listed in Table S1 Table. The 4 biogeographic regions are represented by the colors indicated in the reference key. Map built with QGIS 3.1 Geographic Information System. Open Source Geospatial Foundation Project (http://qgis.osgeo.org).

The genus *Orthohantavirus* (family *Hantaviridae*) [1] includes zoonotic species of RNA virus found in Eurasia, the Americas, and Africa; associated with mammals species of rodents bats and shrews [2]. Rodent borne hantaviruses are the etiologic agents causing two human diseases: hemorrhagic fever with renal syndrome (HFRS), which is transmitted by rodent subfamilies Arvicolinae and Murinae in Euroasia; and the hantavirus pulmonary syndrome (HPS) transmitted by the subfamilies Neotominae and Sigmodontinae in North and South America [3, 4]. Fatality rates of HPS can reach up to 35%–50% in South America [5]. In Argentina, there are 4 endemic regions of HPS: northwestern, northeastern, central and southwestern (Fig 1). The mortality rate varies depending on the endemic region. The overall mortality rate in Argentina during the period 1995–2017 ranged from 21.4% to 25.8%. The highest case-fatality rate (40.5%) occurred in the southwestern endemic region, which is shared with southern Chile [6]. The northwestern endemic region has the lowest mortality rate (15%–17%) but the highest prevalence, near 50% of all reported cases in the country. The main causative agent of HPS in Argentina is the *Andes orthohantavirus* (ANDV). Several variants or genotypes of this viral species were identified. In Northwestern Argentina, the Oran virus (ORNV) is the main circulating variant associated with HPS, but the Bermejo (BEMV) variant and *Laguna Negra orthohantavirus* (LANV) were also identified in human cases [7–9].

The transmission to humans occurs mainly via inhalation of aerosolized excreta from infected rodents, although cases of human to human transmission have been demonstrated in Argentina and Chile [10–13]. Several risk factors have been identified, such as outdoor rural work, recreational activities, deforestation, non-aerated buildings, and virus survival out of their hosts [6, 14,15]. But the epidemiology of human HPS is necessarily linked to the geographic distribution and ecology of the rodent host. Climatic factors are environmental regulators of rodent-borne virus prevalence and transmission rates through their effect on reproductive success in the host population [16]. Because hantaviruses are transmitted horizontally, interaction among individuals is essential to spread the infection in the rodent population; thus changes in rodent density may affect odds of virus spillover into the human population [16–20]. The climate has a strong influence on hantavirus reservoir population dynamics. Particularly the precipitation and the temperature regulates primary production of food resources as well as survival and reproduction of rodent populations [16–23]. These studies showed how climatic variables influencing rodent population dynamics may be used as an indicator for the risk of hantavirus transmissions. Studies reporting environmental correlates of human hantavirus infections in Argentina found factors such as vegetation cover [24, 25], evapotranspiration and human demography [26], rainfall and temperature [25, 27], in addition to rodent distribution [24–27] as explanatory variables. Longitudinal studies on rodents host infections in central [28–30] and south Argentina [31–33] reported a seasonal pattern for rodent density and viral antibody prevalence, although generally not synchronic. Additionally, a higher abundance of rodent reservoirs was associated with warm and rainy weather in central [34, 35] and southern Argentina [36]. However, the relationship between climatic variability and the transmission of hantavirus has not been fully explored, particularly in northwestern Argentina, where the highest HPS prevalence of the country occurs.

In this paper, we investigate human hantavirus infection temporal dynamics related to rainfall and temperature in Northwestern Argentina endemic region for the 1997–2017 period. We test the trophic cascade hypothesis, which states that outbreaks of hantavirus disease are preceded by events of high rainfall and temperatures, which increases resource input for rodents host and the consequent increase in rodent abundance and viral transmission. We aim to move forward on the control and prevention of this disease through an early warning system of potential hantavirus outbreaks based on these ecologically relevant climatic factors.

## Materials and methods

### Study site

Argentina has a great latitudinal extension (ca. 3,700Km) from the tropic of Capricornia (ca. 22° latitude S) southward to the uttermost continental land of America (ca. 55° latitude S). The country also has a large elevation gradient, from sea level in the Atlantic coast up to 6960 m above sea level in the Andes, where the highest summit of America (the Aconcagua) is located. This setting determines a variety of bioclimatic regions, ranging from rainy tropical forest and frosty temperate forest to savannas, grasslands and, deserts. In Northwestern Argentina, most of these biomes are present: 1) savannas-like semi-arid woodlands (Chaco biogeographic province) in the eastern lowlands; 2) montane rainforest, montane temperate forest, and grasslands follow one another on a steep elevation gradient on eastern Andes slopes (Yungas biogeographic province); 3) the western rain shadow slopes and valleys in Northwest Argentinean Andes are xeric scrub deserts (Monte biogeographic province); 4) on the mountain tops above 4000 m.a.s.l. frosty highland steppes occur (Puna biogeographic province). Hantavirus in Argentina occur mainly in humid biomes [14,37]. In northwestern Argentina, the majority of HPS cases occur in the northernmost Yungas rainforest, an area that has been identified as a discrete biogeographic unit based on rodent endemism [38]. Our study area includes part of 2 political provinces of Argentina: Salta and Jujuy (Fig 1B). Within this area, 2 orthohantavirus species were identified: *Laguna Negra orthohantavirus* (LNV), carried by *Calomys laucha*, *Calomys callosus*; and 2 variants of *Andes orthohantavirus* (ANDV): Oran (ORNV) and Bermejo (BEMV) virus carried by *Oligoryzomys chacoensis* and *Oligoryzomys flavescens* respectively.

### Climatic data

The climate in northwestern Argentina is seasonal with a monsoonal precipitation regimen concentrated during summertime of the Southern Hemisphere. Due to the orographic effect of the Andes, moisture is partly released in the form of intense rainfall at the eastern flanks of the tropical/subtropical Andes. This results in a rainfall gradient between the humid low-elevation (1200 mm/yr) close to the mountain (Yungas) and eastern semi-arid (Chaco) plain regions eastward (500 mm/yr). The mean annual temperature in our study area is 21°C, with minimum values of -2°C in Oran (Northern Salta) and -5°C in Perico (southern Jujuy) and maximum of 43°C and 41°C respectively, during the studied period (Fig 1B). For the statistical analysis, we obtained monthly climatic data of near-surface mean temperatures and total precipitation from the Climate Research Unit (CRU TS 4.01, http://www.cru.uea.ac.uk/) at the University of East Anglia, UK. This dataset corresponds to interpolated data of instrumental records from a dense network of local meteorological stations (Table S2 Table) with homogeneity tests and scaled to a 0.5-degree square network [39]. We used the gridded monthly average temperature and total precipitation centered at 24.25°S; 64.75°W (Yuto city, Jujuy province; Fig 1B).

### Hantavirus pulmonary syndrome cases

A patient who resides in the Northwest region presenting, in the prodromic phase, persistent fever (>48hs), headache, myalgias and/or gastrointestinal manifestations (abdominal pain, vomiting and/or diarrhea) and adding respiratory compromise in the advanced stage of the illness was defined a suspected HPS case. For this study, we included all suspected cases that were confirmed by laboratory tests according to the diagnostic algorithm of the Hantavirus National Reference Laboratory [14]. Cases were laboratory-confirmed by the presence of both IgM and IgG antibodies; cases with IgM titers but not IgG were confirmed verifying IgG seroconversion in second samples and/or by viral RNA detection by reverse transcription-quantitative polymerase chain reaction (RTqPCR) and/or RT-PCR followed by nucleotide sequencing [14]. The confirmation was done at the National Reference Laboratory from The National Institute of Infectious Disease "Instituto Nacional de Enfermedades Infecciosas Dr. C. G. Malbrán " (INEI) of the Administración Nacional de Laboratorio e Institutos de Salud (ANLIS). Because HPS is a reportable disease, we also considered cases that were reported through the National Health Surveillance System (SIVILA/SISA) by independent laboratories. However, cases reported through SIVILA by independent laboratories performing diagnostic tests not validated by the National Reference Laboratory were not taken into account for this study. We compiled HPS cases from northwestern Argentina that occurred from summer 1997 to summer 2017.

### Ethics statement

Data reported by the National Health Surveillance System are completely anonymous. Additionally, National Institute for Infectious Diseases "Instituto Nacional de Enfermedades Infecciosas" (INEI) and the National Health Surveillance System (SIVILA/SISA) follows the principle of ethics in research for the collection of data-keeping with the Declaration of Helsinki on human study and the Research in Human Beings Guide of the National Ministry of Health.

### Statistical analysis

Given the climatic regimen in the studied area, we first grouped HPS cases and climatic data into 6-month periods representing the rainy season (October-March) and the dry season (April-September). To analyze the relationship in a finer time scale, we also grouped the number of hantavirus human infections and climatic data into a 3 and 2-month period. We used log10 transformed number of HPS cases to stabilize the variance. Alternatively, we removed outliers (2 observations for the biannual data arrangement; and 5 observation for the quarterly and bimestrial data arrangement (3 in 2006/2007 and 2 in 2015). We standardized all data to allow comparison of different magnitude scores and applied 1 seasonal difference to achieve stationarity, checked using the Augmented Dickey-Fuller Test. We used dynamic regression to evaluate the relationship of HPS prevalence with rainfall and temperature. This technique involves a regression model that relates to delayed values of the explanatory variables (rainfall and temperature) with the response variable (HPS cases). Time series data frequently exhibit correlation with its own past values (autocorrelation), leading a standard regression method to return incorrect coefficients and invalid significance tests. To overcome this problem, the autocorrelation is accounted for by a specific error term which includes estimated coefficients for the autoregressive (AR) and moving average (MA) components. These coefficients represent the contribution of the previous values (AR) and innovations (MA) to the present observed value. The number past values (lags) to be included are specified by the order "p" for the AR process and "q" for MA, denoted by (p, d, q). The "d" represents the degree of differencing (subtracting its current and previous values d times) used to stabilize the series when the stationarity assumption is violated. This is known as regression with ARIMA errors [40]. The regression part and the ARIMA part of the model are computed separately and then both results are combined. We built models for all possible combinations of lagged rainfall and temperature from the previous season (t-1) to a delayed time lag of 6 periods (t-6), equivalent to 3 years for the biannual data arrangement, and of 8 periods (t-8) equivalent to the previous 2 years for the quarterly data arrangement and 1 year for the bimestrial data. We used the corrected Akaike Information Criterion (AICc) for model selection and checked for random residuals. The model with the lowest ACIc value was selected as the best one, and the differences in the scores between each model and the best one (ΔAICc) was calculated. Models with ΔAICc<2 were considered equally parsimonious and essentially as good as the best model [41]. We also calculated the adjusted coefficient of determination (R^2^ adj) as a measure of model fit and the Root Mean Square Error (RMSE) as a measure of the model forecast accuracy for the training set one step ahead. All statistical analyses were performed in R using the forecast package [42].

## Results

**Fig 2:**
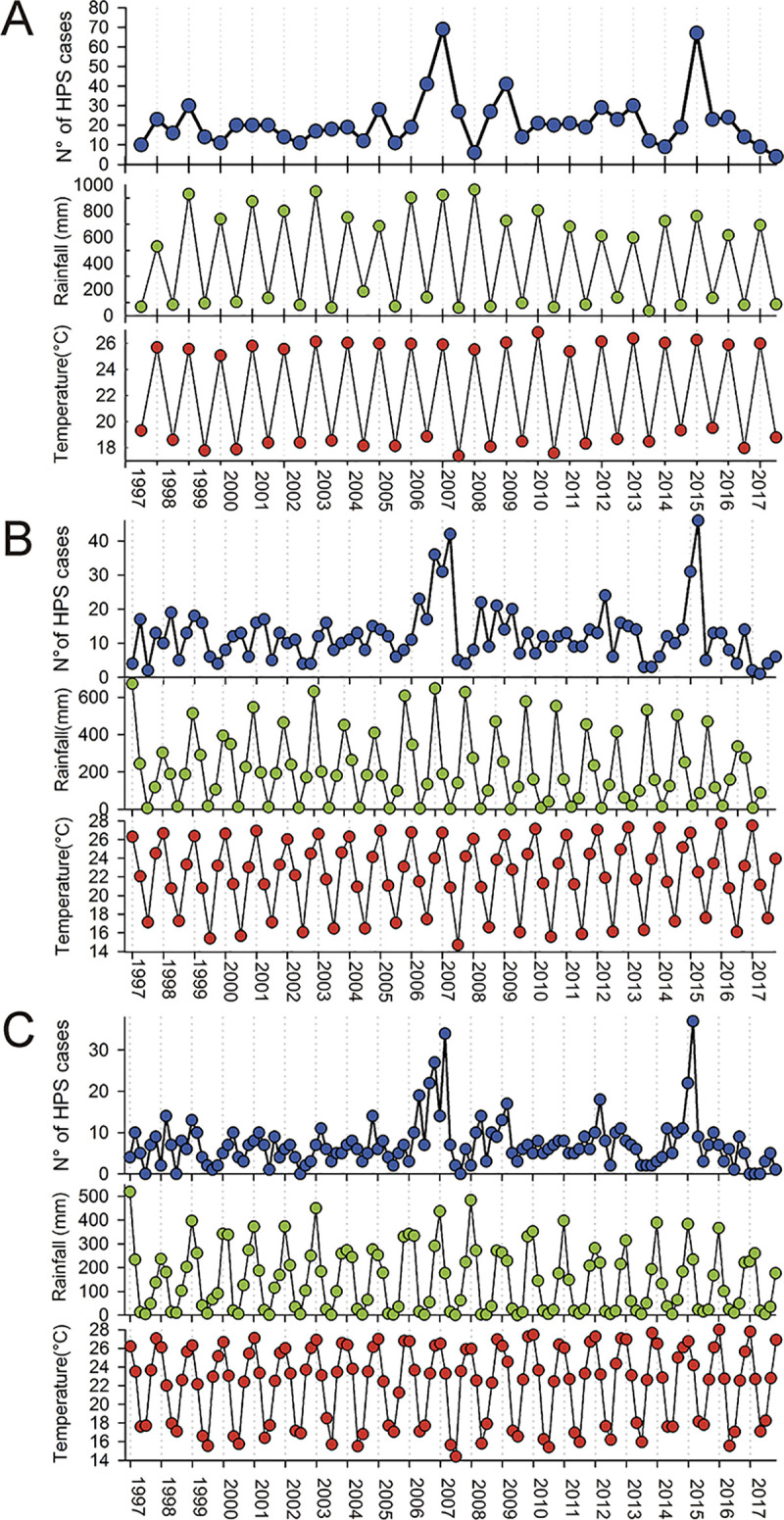
Hantavirus pulmonary syndrome (HPS) cases and climate variability for the 20 years analyzed. Variation in the number of HPS cases for Salta and Jujuy provinces (Northwestern Argentina), and total rainfall and mean temperature estimated for (A) biannual data arrangement, (B) quarterly and (C) bimestrial data arrangement. Climatic variables were estimated for Yuto city (Jujuy) during the 20 years analyzed starting in the summer of 1997.

We recorded 902 HPS cases for the 20 years analyzed, of which 602 were in Salta province and 300 in Jujuy province (Fig 1B). The accumulated number of HPS cases for each time interval, as well as the average and range of rainfall and temperature, are summarized in Table 1. Variation in HPS cases, rainfall, and temperature for the studied period are shown in Fig 2. There were 2 clear abrupt increments in HPS case numbers from spring 2006 through autumn 2007 and during the summer and autumn in 2015.

**Table 1:** Hantavirus pulmonary syndrome (HPS) cases and climatic variables. The accumulated number of HPS cases and climatic variation in rainfall and temperature for biannual, quarterly, and bimestrial data arrangement.

Biannual	HPS cases	Mean rainfall mm	Rainfall range	Mean temperature C°	Temperature range
Apr-Sep	373	92.2	36.5–184.4	18.4	13.2–28.8
Oct-Mar	528	765.4	528.7–964.4	25.9	14. -28.5
Quarterly					
Jul-Sep	112	4.5	0–38.8	18.3	13.2–23.2
Oct-Dic	247	89.8	12.6–267.5	25.8	23–28.8
Jan-Mar	281	167.1	51.6–313.8	26	23–28.5
Apr-Jun	261	25.9	0.3–122.6	21.4	14.0–23.6
Bimestrial					
Jul-Aug	58	4.3	0–14.7	16.7	13.2–18.0
Sep-Oct	142	57.4	12.8–126.1	23	18.9–26.7
Nov-Dic	160	221.1	90.7–331.4	26.3	23.7–28.8
Jan-Feb	154	354.7	225.5–518.8	26.7	24.7–28.5
Mar-Apr	238	205.9	58.9–338.9	23.1	19.5–26.2
May-Jun	149	1.9	2.7–39.8	16.9	14–20

The results of model comparison for hantavirus infections dynamics related to the rainfall and the temperature in Northern Argentina are listed in Table 2. The results of model comparison when the 2006/7 and 2015 outbreaks were eliminated are listed in Table S3 Table.

**Table 2:** Model selection. List of best models for different combinations of lagged rainfall, temperature, and ARIMA error according to the corrected Akaike Information Criterion (AICc).

Biannual Models	AICc	ΔAICCc	RMSE	R^2^_adj_
Rainfall(t-1), Temperature(t-1), AR 2	97.74	0	0.87	0.69
Rainfall(t-1, t-2), Temperature(t-2) AR 2	100.09	2.39	0.86	0.68
Rainfall(t-1), Temperature(t-0) AR 2	101.29	3.59	0.91	0.64
Rainfall(t-1, t-2), Temperature(t-1, t-2), AR 2	101.82	4.08	0.84	0.70
Rainfall (t-1) AR 4	102.09	4.35	0.86	0.68
Quarterly Models				
Rainfall(t-1), Temperature(t-2), AR 1, MA 4	206.1	0	0.88	0.71
Rainfall(t-1, t-2), Temperature(t-2), AR 4	208.5	2.39	0. 90	0.69
Rainfall(t-1), Temperature(t-0, t-2), AR 4	208.7	2.59	0.88	0.70
Rainfall(t-1), Temperature(t-1), AR 1 MA 4	210.0	3.99	0.79	0.69
Rainfall(t-1), Temperature(t-4), AR 1 MA 4	210.0	3.99	0.78	0.70
Rainfall(t-2), Temperature(t-2), AR 1 MA 5	210.1	4.00	0.78	0.69
Rainfall(t-1), Temperature(t-3), AR1 MA 4	211.2	5.09	0.79	0.69
Bimestrial Models				
Rainfall(t-1, t-2), Temperature(t-1, t-2), MA 5	284.7	0	0.78	0.61
Rainfall(t-1, t-2), Temperature(t-2), AR 1, MA 2	285.8	1.06	0.81	0.58
Rainfall(t-0, t-1, t-2), Temperature(t-0, t-1, t-2), AR 1, MA 2	286.5	1.71	0.78	0.60
Rainfall(t-1), Temperature(t-2) AR 1, MA 2	289.5	4.79	0.83	0.56
Temperature(t-1), MA 3	291.4	6.68	0.84	0.54

**Fig 3:**
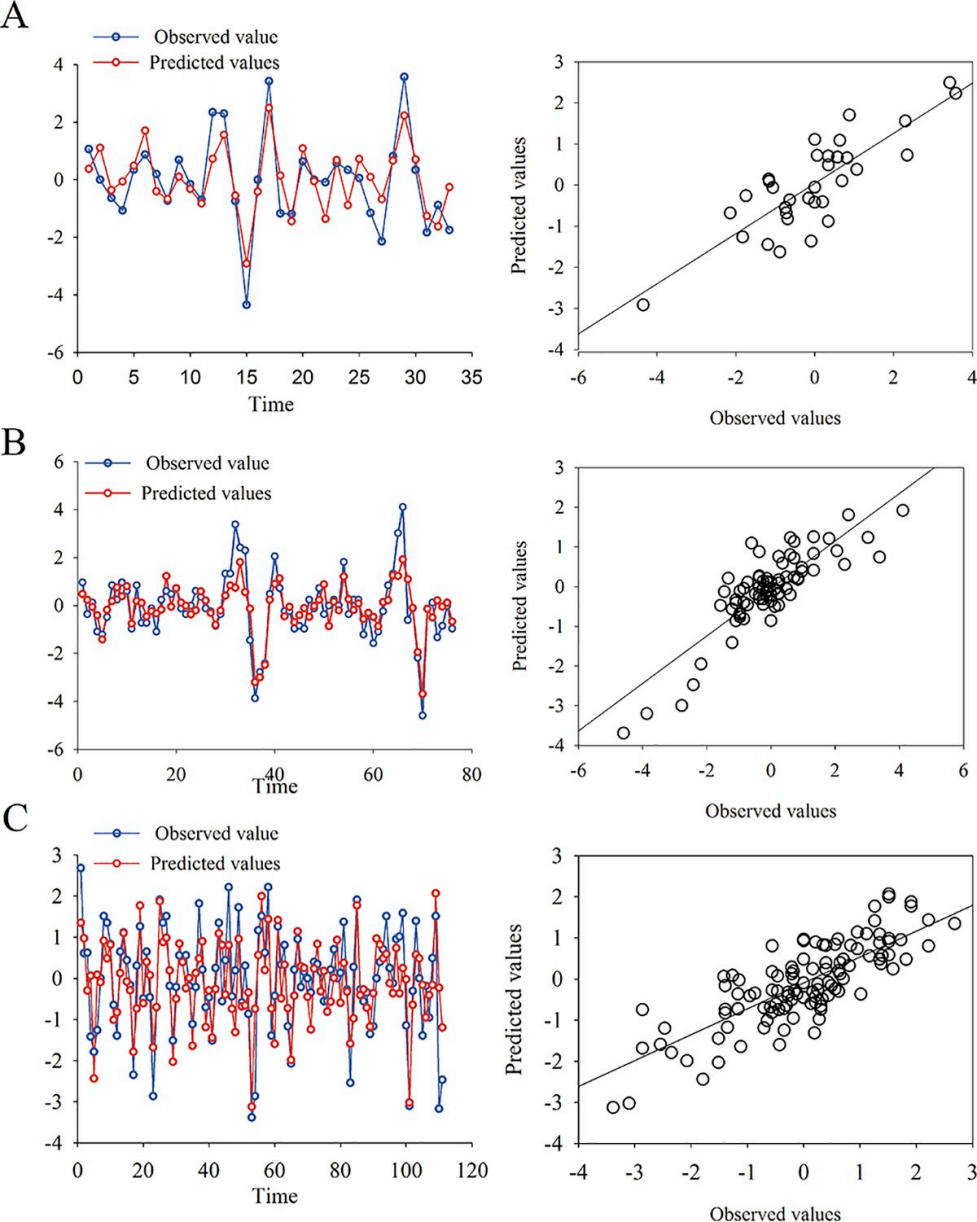
Plots of observed and fitted hantavirus infections. Observed values versus the predicted by the selected model for hantavirus infections in northwestern Argentina. (A) Biannual data arrangement with rainfall (t-1) and temperature (t-1) as explanatory variables. (B) Quarterly data arrangement with rainfall (t-1) and temperature (t-2) as explanatory variables. (C) Bimestrial data arrangement with rainfall (t-1, t-2) and temperature (t-1, t-2) as explanatory variables.

For the biannual data arrangement, a single model was the most parsimonious (AICc = 97.74) which explained 69% of the variation in HPS cases (Table 2, Fig 3A). This model included the rainfall and the temperature in the previous 6 months (1 lagged period) as explanatory variables, both positively related to HPS infections and statistically significant (Table 3). The ARIMA model accounting for serial dependence was a second-order autoregressive possess. Ljung-Box test (Q* = 2.25, df = 3, p-value = 0. 52) showed there is no significant structure remaining in model residuals. The 3 following most parsimonious models were equivalent (ΔAICc range 2.9–4.0) and should be considered relevant models as well (Table 2). All these models included a combination of the rainfall with 1 and 2 lagged periods and the temperature with 0, 1, or 2 lagged periods as explanatory variables (see Table 3). When the 2 outbreaks were removed from the data set, 4 models appeared as equally parsimonious which explained 35 to 50% of the variation in HPS cases (Table S3 Table). These models included a direct relationship with the rainfall for 1 or 2 delayed periods and temperature with none or 1 delayed period. Additionally, we found a significant inverse relationship for hantavirus infections with 2 or 3 lagged periods of temperature for these models (Table S3 Table).

**Table 3:** Coefficients estimated for the best-fitting model of hantavirus infections and the two explanatory climatic variables in northwestern Argentina. Only significant coefficients are listed, all estimated for standardized z- values in first seasonal difference and log10 transformed number of hantavirus infections. AR: Autoregressive and MA: Moving average component of the ARIMA error term component.

Biannual Model			
Rainfall(t-1), Temperature (t-1)	Estimated	Standard error	p-value
Rainfall(t-1)	1.51	0.50	>0.01
Temperature (t-1)	3.85	1.09	>0.01
AR 1	0.29	0.13	>0.01
AR 2	-0.66	0.12	>0.01
Quarterly Model			
Rainfall(t-1), Temperature (t-2)			
Rainfall (t-1)	0.62	0. 23	>0.01
Temperature (t-2)	1.00	0.50	>0.05
AR 1	0.38	0.12	>0.02
MA 4	-1.00	0.11	>0.01
Bimestrial Models			
Rainfall(t-1, t-2), Temperature(t-1, t-2)			
Rainfall(t-1)	0.34	0.15	>0.05
Rainfall(t-2)	0.30	0.13	>0.05
Temperature (t-1)	0.30	0.12	>0.01
Temperature (t-2)	-0.58	0.16	>0.01
MA 1	0.48	0.11	>0.01
MA 2	-0.66	0.14	>0.01
MA 3	-0.26	0.13	>0.05
MA 5	-0.25	0.11	>0.05
Rainfall(t-1, t-2), Temperature(t-2)			
Rainfall(t-1)	0.57	0.11	>0.01
Rainfall(t-2)	0.32	0.13	>0.01
Temperature (t-2)	-0.61	0.16	>0.01
AR 1	0.51	0.10	>0.01
MA 2	-0.90	0.06	>0.01
Rain(t-0,-1, -2), Temperature (t-0, -1, -2)			
Rainfall(t-1)	0.45	0.18	>0.01
Rainfall(t-2)	0.42	0.18	>0.05
Temperature (t-1)	0.16	0.18	>0.05
Temperature (t-2)	-0.70	0.23	>0.05
AR 1	0.54	0.10	>0.01
MA 2	-0.91	0.06	>0.01

For the quarterly data arrangement, the model with the lowest AICc (206.11) explained 71% of the variation in hantavirus infections (Table 2, Fig 3B). The best model included the rainfall in the previous period and the temperature with 2 periods of delay as explanatory variables. Both positively related to hantavirus infections and statistically significant (Table 3). The ARIMA model of the error term accounted for the autocorrelation in the time series with a 1st order AR component and a 4th order MA component. The estimated coefficients for the model are listed in Table 3. Ljung-Box test (Q* = 5.38, df = 3, p-value = 0.15) showed there is no significant structure remaining in residuals of the model. All the following most parsimonious models also included a positive relationship of mean rainfall with 1or 2 lagged period and mean temperature with 1 to 3 lagged periods (Table 3). When outbreaks were not taken into account, 2 best models appeared as equally simplest models, explaining 50 to 53% of hantavirus transmission. In both models, HPS cases were positively related to rainfall for 1 and 3 lagged period, but negatively related to temperature for the same time intervals (Table S3 Table).

For the bimestrial data arrangement 3 models were equally parsimonious (ΔAICc<2) accounting for 58% to 61% of the variance in HPS cases (Fig 3C). The first model (AICc = 284.7) included the rainfall and the temperature with 1 and 2 lagged periods respectively as explanatory variables. A fifth-order moving average term accounted for serial dependence in this model (Ljung-Box test: Q* = 7.5, df = 3, p-value = 0.07). The following simpler model (AICc = 285.8) was similar, but only included the second lagged period for the temperature, and a first-order autoregressive and second-order moving average component in the ARIMA error term (Ljung-Box test: Q* = 7.4657, df = 4, p-value = 0.1132). The third one (AICc = 286.5) included the present and two lagged periods for both the rainfall and the temperature for both. A first-order autoregressive and second-order moving average error term accounted for serial dependence in this model (Ljung-Box test: Q* = 7.24, df = 3, p-value = 0.07). However, the relationship between HPS cases and both climatic variables without temporal delay was not significant. On the contrary, for all these models HPS cases were positively and significantly related to the rainfall for all past time lags as well as the temperature for 1 delayed period but inversely related to temperature for the second lagged period (Table 3). When outliers were eliminated a similar couple of selected models explained for 58 and 55% of hantavirus infections variability. Identically, both models indicate a positive relation of HPS cases with the rainfall and a negative relations with the second lagged period for temperature (Table S3 Table).

## Discussion

In this study, we investigate the relationship between human hantavirus infections and climatic variability for 20 years in northwestern Argentina. We divided our data into 3 different time periods to allow annual and shorter time delays to be observed, and found a significant association between hantavirus transmission and lagged rainfall and temperature with a delay of 2 to 6 months. Additionally, we removed extreme values of 2006 and 2015 outbreaks, to look for the underlying dynamic in hantavirus transmission and found a very similar set of candidate models. These results are valuable for the development of an early warning tool for public health policies based on climatic parameters. However, the risk of human hantavirus infection is determined by multiple level interactions of environmental, ecological, behavioral, and anthropological factors that may affect the rodent host population, the viral transmission from one host to another, and finally, the spillover to humans [43]. Notwithstanding our study revealed the importance of climatic variability in determining the dynamic of hantavirus disease transmission. In this sense, our results are consistent with the reports of several researchers worldwide [see reviews 44–46].

Our analyses showed a significant positive relationship between human hantavirus infections and the delayed rainfall for all selected models and lagged periods (see Table 3). The connection between high average precipitation and HPS outbreaks has been interpreted as the consequence of an increased resource input for rodents host via plant primary productivity, raising the carrying capacity of the ecosystem and the consequent increase in rodent abundance and viral transmission [19, 21]. However, the reservoir host and pathogen dynamics are complex and outbreaks depend on several factors, such as a critical host population density necessary to maintain infections and enough time to sustain a chain of transmission after an increase in the carrying capacity [47]. Although low antibody prevalence was frequently reported in periods of high rodent density, when juveniles not yet infected individuals prevailed in the population [28, 30]; the highest absolute numbers of antibody-positive animals were associated with the highest population densities, thus the relatively higher risk to humans in central Argentina [34, 35, 37]. Despite this complexity, the rainfall and/or rodent abundance has been associated with an increased risk of viral transmission to human populations even without any knowledge about hantavirus dynamics in the host populations [17–19, 21]. Unfortunately, there are no available data on the temporal variation in rodent abundance from northwestern Argentina. However, a meta-analysis on rodent outbreaks "ratadas" in South America showed that most rodent species respond quite soon to rainfall peaks, about 3 to 6 months after the rains [48]. Similarly, the accumulated precipitations in the previous months positively affected rodent abundance in central Argentina [34, 35]. The present study corroborates the significance of the rainfall in the transmission of hantavirus to human populations, probably related to a bottom-up trophic cascade effects on potential rodent hosts. Notoriously, 2 geographical outliers of hantavirus infections occurred by the time of both outbreaks. One in 2006 close to the eastern border of Salta, in the neighbor Formosa province [6]. The other one in 2016 was the southernmost HPS case of northwestern Argentina in Tucumán province [49] (Fig 1B). This suggests a probable broad-scale environmental influence on ecosystems productivity.

Our results also indicate that the temperature has a significant relationship with hantavirus transmission dynamics. All our bests models included a positive relationship of HPS incidence with delayed temperature (Table 3). The outbreaks in 2006 and 2015 were presided by increasing winters temperatures (see Fig 2). As an environmental regulator, the temperature may also affect the transmission and persistence of hantavirus at the rodent population level. Because of the horizontal transmission of hantavirus in the reservoir populations, the longevity of even a small proportion of the host population in cold months may provide a trans-seasonal mechanism for virus persistence [29]. Empirical studies in central Argentina found the highest rodent abundances were preceded by warmer winters [34, 50]; the larger the overwintered cohort the higher of population peaks [50]. Because seasonal breeding in most mammals is related to the physiological stress associated with thermoregulatory energetic demand that produces reproductive suppression to keep up energy balance, warm winter temperatures may modify the reproductive period to the degree of winter breeding [51, 52]. This hypothesis should be tested by rodent population dynamics data from nonwestern Argentina.

**Fig 4:**
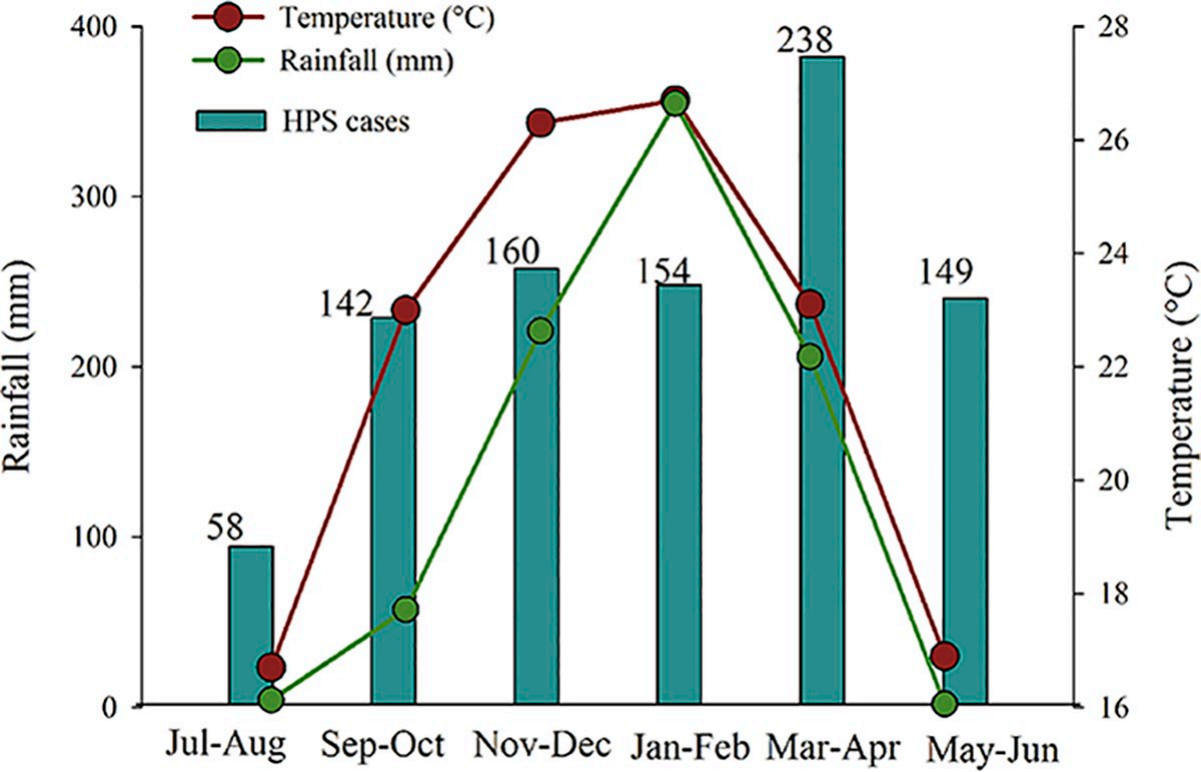
Bimestrial distribution of hantavirus pulmonary syndrome (HPS) cases, mean temperature and rainfall for the (1997–2017). Numbers above bars indicate accumulated HPS cases, red circles indicate mean temperature, and green circles indicate mean rainfall.

Remarkable, our models revealed somehow contradictory findings on the relationship of temperature with hantavirus transmission. For the bimestrial data arrangement, the 3 bests models included a significant inverse relationship between hantavirus infections and the temperature 4 months before (see Table 3). We found an identical relationship for hantavirus infections with the lagged temperature for the bimestrial data without the outliers (Table S3 Table). In our study site the highest HPS incidence occurred in March-April (late summer early fall). During the previous 4 month-period, in November-December (late spring) the temperature is as high as in January—February (summer) but with much less rainfall (Fig 4). A possible explanation was that the high temperature makes unfavorable the environment for virus survival, together with a reduced frequency of rodents and rodent-human contacts [53]. Experiments with Puumala (PUUV) and Tula (TULV) viruses demonstrated that low temperatures prolonged virus survival outside the host, which still infective even without direct contact between rodents, or between rodents and humans [54]. However, evidence for a role of low temperatures on the indirect Andes virus (ANDV) transmission in rodents reservoirs still not conclusive, given that infection was not observed between wire mesh-separated animals nor from excrement-tainted bedding in an experimental study on wild sigmodontine rodents [55]. Similarly, the role of low temperatures in the transmission to humans appears as not plausibly given the 4-month time lag indicated by our models and the estimated incubation period of 9 to 40 days for this disease [6, 56]. Noteworthy, studies on Neotropical rodents showed that daily activity decreases with rising temperature [57, 58], but increase when combined with rain [59]. Note that all our bimestrial best models also included a significant positive relationship with rainfall in the second lagged period (Table 3). It should be also noted that effects of heat stress range from disruptions in spermatogenesis and oocyte development, oocyte maturation, early embryonic development, fetal growth, and lactation [60]. Thus, in addition to a heat-induced reduction in rodent activities, which could reduce potential infectious contact between rodents, a heat-induced delayed reproductive season may also be considered as a hypothesis to be explored. Although the mechanism should be deeply investigated, our models suggest a strong inverse relationship of the temperature, and/or direct evaporative cooling effect of rainfall, for the transmission of hantavirus in northwestern Argentina (see Table 3).

**Fig 5:**
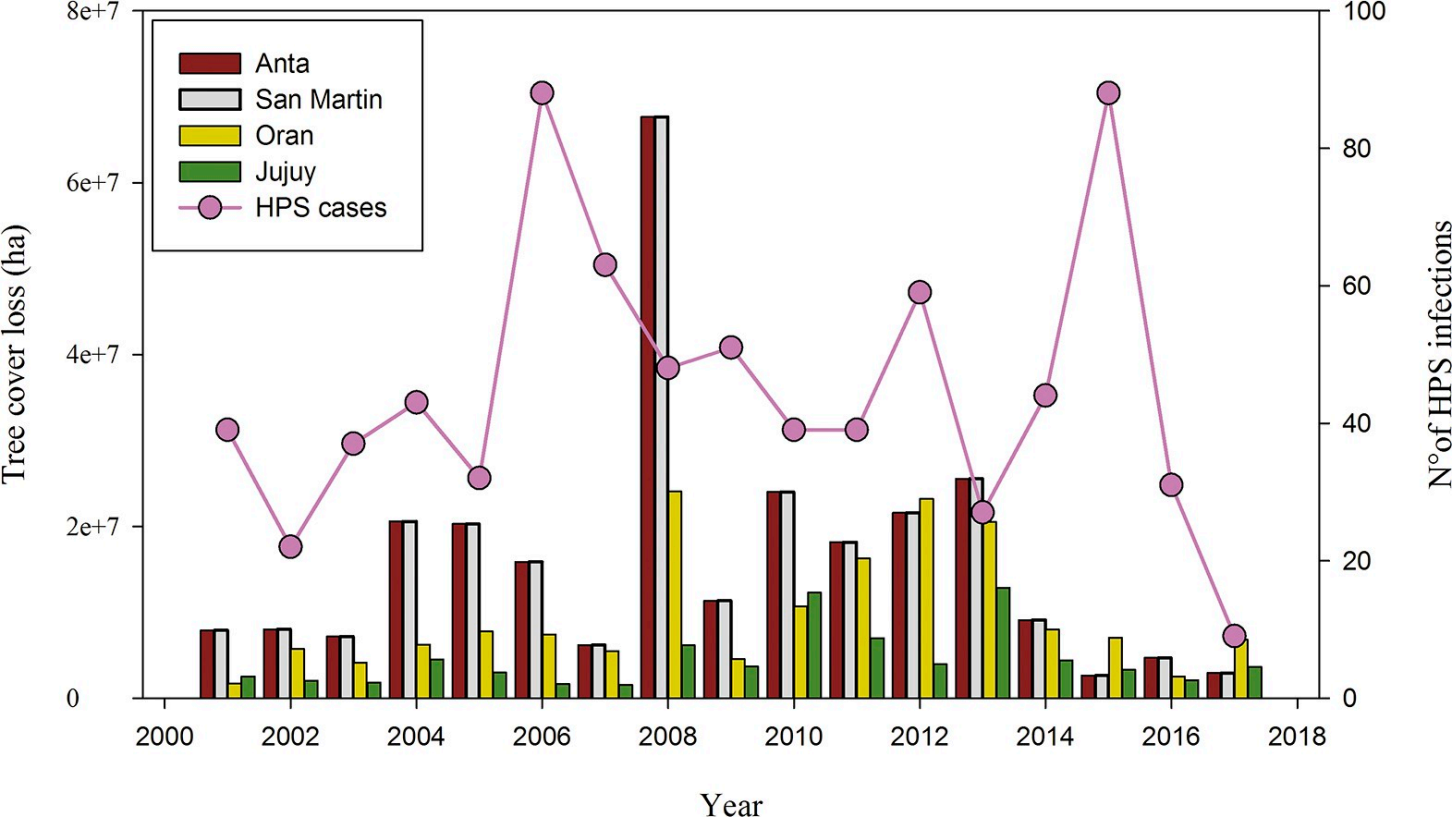
Hantavirus pulmonary syndrome (HPS) cases and tree cover loss in Northwestern Argentina. The circles indicate the annual variations in HPS cases whereas the bars indicate year-by-year tree cover loss (millions of hectares) for Jujuy Province (green) and 3 northeastern Departments of Salta province: Anta (red), San Martín (pink), Oran (yellow). Note that tree cover loss does not need to be human-caused. Source: Global Forest Watch. “Tree Cover Loss in Salta and Jujuy, Argentina”. Accessed on 13/05/2020 from www.globalforestwatch.org.

Beyond the ecological response of the rodent hosts to climatic variability, human activities may be directly related to hantavirus infections via potential contact with infected rodents. In northwestern Argentina, the majority (64.2%) of the cases were occupational affecting mainly rural workers [14]. The climate can affect the dynamics of hantavirus infections through multiple pathways. For instance, the activities in the forest harvesting industry, which involve several days' work in precarious camps, are almost null during the summer due to the heavy rainfall. Similarly, the horticulture is an intensive labor activity with a significant number of temporary workers, especially at harvest time from late summer to late autumn. Human contact with infected rodents is likely to be higher during periods of intense field labor, therefore contributing to the seasonal dynamics in humans hantavirus infections. Additionally, the agriculture frontier rapidly expanded since the 2000s associated with soybean production [61]. The outbreak in 2006–2007 may be related to an accelerated deforestation process driven by Argentina’s National Congress treatment of the “Forest Law” (National Low N° 26331), to regulate the management and conservation of native forests, which was finally approved in November 2007. However, the higher deforestation rates were in the Chaco during 2003–2005 [61, 62] and with a marked increase in 2008 (Fig 5). On the contrary, most HPS cases occurred in the Yungas Forest with a few cases in the Chaco (see Fig 1) [6]. It should be noted, however, a possible geographical bias toward the urban centers, which are located in the Yungas Forest, given that many reports only were recorded at the departmental geographical level. Furthermore, there is no evidence of an increase in tree loss cover and the 2015 outbreak. However, an assessment of the relationship between deforestation and hantavirus infections should be specifically designed.

The limitations of our data and analysis should be also be noted. First, climatic and HPS data were pooled into a single interpolated square of 0.50°. There is a clear latitudinal gradient in hantavirus infections which is lost by our approach. Second, extreme climatic events, which may have important biological and social consequences, were overlooked due to the use of averaged temperature; and the few rainfall meteorological stations covering the entire studied time period. Third, the response variable accuracy is a limitation in this study. Several misdiagnoses or incomplete diagnoses of HPS, as well as data obtained through independent laboratories and provincial laboratories without the supervision of the National Reference Laboratories could not be included in this analysis. Finally, the prevalence of hantavirus antibodies in the human population in the studied area averaged 6.5% [63,64] denoting frequent and close contact with host rodents. The number of HPS cases might go unreported because of milder clinical symptoms. It is likely that all these limitations influence the precision of our results.

Hantavirus pulmonary syndrome is a serious disease in northwestern Argentina. Most of the reported cases (75%) developed severe respiratory insufficiency with hemodynamic compromise, of which 30% required mechanical ventilation and 15% developed a refractory to treatment hemodynamic compromise with a fatal outcome [14]. Particularly in the studied region, there is a great proportion of the population with high levels of social vulnerability. In Salta province 45% of the population is below the poverty line and 37% in Jujuy province [65]. The rural areas, where most infections occur, receive scant public investment on social infrastructures, education, health, and individuals are least likely to have appropriate healthcare. Since there are no vaccines currently available nor specific therapeutic treatments, prevention of hantavirus infection involves mainly environmental management practices and educational campaigns. Therefore, our models are valuable for the planning and implementation of public health prevention campaigns. In this sense, our results provide a framework to advance on an early warning of potential hantavirus outbreaks based on the significant relationship between delayed climatic variables and the hantavirus transmission. However, a specifically designed HPS surveillance program, including a standardized data repot of HPS through Hantavirus National Laboratory Network would enhance surveillance in Argentina. Also, surveys on rodent population and virus dynamics will lead to a more accurate models to forecast HPS outbreaks in northwestern Argentina.

## Supporting information

S1 Table List of localities of known occurrence of HPS cases in Northwestern Argentina. (DOCX)

S2 Table Station list, location, and time cover used by Climatic Research Unit TS 4.02 in the gridded temperature and precipitation data. (DOCX)

S3 Table List of best models for different combinations of lagged rainfall, temperature and ARIMA error according to corrected Akaike Information Criterion (AICc) for HPS infections without outliers. (DOCX)
