# Supplementary material for: Hantavirus pulmonary syndrome outbreaks associated with climate variability in Northwestern Argentina, 1997–2017
Source: PLoS Negl Trop Dis. 2020 Nov 30;14(11):e0008786. doi: 10.1371/journal.pntd.0008786 (PMC7728390; doi:10.1371/journal.pntd.0008786)
Supplement: S1 Table — (DOCX) [file pntd.0008786.s001.docx]

S1 Table. List of localities of known occurrence of Hantavirus Pulmonary Syndrome in Northwestern Argentina.

| Locality | Department | Province | Latitude | Longitude |
| --- | --- | --- | --- | --- |
| Salvador Maza | SAN MARTIN | SALTA | -22.08884 | -63.69971 |
| Aguaray | SAN MARTIN | SALTA | -22.24178 | -63.75115 |
| Los Toldos | SANTA VICTORIA | SALTA | -22.31182 | -64.66487 |
| Madrejones | SAN MARTIN | SALTA | -22.33733 | -64.09608 |
| Virgen de la Ppeña | SAN MARTIN | SALTA | -22.47844 | -63.80669 |
| Tartagal | SAN MARTIN | SALTA | -22.53496 | -63.8182 |
| Vespucio | SAN MARTIN | SALTA | -22.59823 | -63.86248 |
| General Mosconi | SAN MARTIN | SALTA | -22.61326 | -63.81612 |
| Angosto del Pescado | SANTA VICTORIA | SALTA | -22.68484 | -64.57653 |
| Aguas Blancas | ORAN | SALTA | -22.72407 | -64.36385 |
| Finca Granados | ORAN | SALTA | -22.85569 | -64.36317 |
| Río Pescado | ORAN | SALTA | -22.87065 | -64.49531 |
| Isla Cañas | IRUYA | SALTA | -22.89499 | -64.67606 |
| Peña Colorado | ORAN | SALTA | -22.90377 | -64.37979 |
| General Ballivian | SAN MARTIN | SALTA | -22.93047 | -63.85804 |
| Villavian | SAN MARTIN | SALTA | -22.94154 | -63.85914 |
| Abra Grande | ORAN | SALTA | -23.01562 | -64.42647 |
| Pasaje Carmelita | ORAN | SALTA | -23.0575 | -64.40805 |
| Finca Rio Blanco | ORAN | SALTA | -23.07447 | -64.3234 |
| San Andrés | ORAN | SALTA | -23.0842 | -64.86704 |
| Río Blanco | ORAN | SALTA | -23.09437 | -64.33014 |
| El Oculto | ORAN | SALTA | -23.10617 | -64.54039 |
| Oran | ORAN | SALTA | -23.11257 | -64.29217 |
| Los Naranjos | IRUYA | SALTA | -23.11757 | -64.67413 |
| El Potrillo | RAMÓN LISTA | FORMOSA | -23.16216 | -62.01535 |
| Río Blanquito | ORAN | SALTA | -23.18485 | -64.68701 |
| Embarcación | SAN MARTIN | SALTA | -23.20382 | -64.08956 |
| Hipólito Yrigoyen | ORAN | SALTA | -23.23454 | -64.26553 |
| San Marín | ORAN | SALTA | -23.26213 | -64.24435 |
| Tabacal | ORAN | SALTA | -23.26496 | -64.25461 |
| Pichanal | ORAN | SALTA | -23.33072 | -64.2498 |
| Colonia Santa Rosa | ORAN | SALTA | -23.37721 | -64.41017 |
| Urundel | ORAN | SALTA | -23.54607 | -64.39475 |
| El talar | SANTA BARBARA | JUJUY | -23.56582 | -64.35865 |
| Yuto | LEDESMA | JUJUY | -23.63509 | -88.2113 |
| Vinalito | SANTA BARBARA | JUJUY | -23.66294 | -64.41754 |
| Caimancito | LEDESMA | JUJUY | -23.73537 | -64.5922 |
| Calilegua | LEDESMA | JUJUY | -23.77175 | -64.76166 |
| Libertador | LEDESMA | JUJUY | -23.80307 | -64.80341 |
| Rivadavia | RIVADAVIA | SALTA | -24.2067 | -62.89515 |
| San Salvador de Jujuy | Dr. M. BELGRANO | JUJUY | -24.21598 | -65.33181 |
| San Pedro | SAN PEDRO | JUJUY | -24.22156 | -64.85225 |
| Palpala | PALPALA | JUJUY | -24.25215 | -65.1378 |
| El Carmen | EL CARMEN | JUJUY | -24.38917 | -65.24339 |
| Finca La Flaca | ANTA | SALTA | -24.4022 | -63.9134 |
| Apolinario Saravia | ANTA | SALTA | -24.42481 | -63.98144 |
| Pampa Blanca | EL CARMEN | JUJUY | -24.5247 | -65.07515 |
| Rio Dorado | ANTA | SALTA | -24.53351 | -64.12416 |
| Las Lajitas | ANTA | SALTA | -24.72478 | -64.17846 |
| General Güemes | GUEMES | SALTA | -24.73095 | -65.02482 |
| San Agustín | CHICOANA | SALTA | -25.00394 | -65.43232 |
| El Quebrachal | ANTA | SALTA | -25.32931 | -64.00303 |
| Yerba Buena | YERBA BUENA | TUCUMAN | -26.79399 | -65.16504 |
