# Supplementary material for: Hantavirus pulmonary syndrome outbreaks associated with climate variability in Northwestern Argentina, 1997–2017
Source: PLoS Negl Trop Dis. 2020 Nov 30;14(11):e0008786. doi: 10.1371/journal.pntd.0008786 (PMC7728390; doi:10.1371/journal.pntd.0008786)
Supplement: S2 Table — (DOCX) [file pntd.0008786.s002.docx]

S2 Table. CRU TS 4.02 Meteorological station list used in the gridded temperature and precipitation data.

| Station list |  |  |  |  |
| --- | --- | --- | --- | --- |
| CRU TS 3.23 station | Sation ID | Latitude | Longitude | Temporal cover |
| *Temperature* |  |  |  |  |
| Jujuy Aero | 8704600 | 24.38S | 65.08W | 1931 - 2017 |
| Salta Aero | 8704700 | 24.85S | 65.48W | 1873 - 2017 |
| Tucumán Aero | 8712100 | 26.85S | 65.10W | 1953 - 2017 |
| Santiago del Estero | 8712900 | 27.77S | 64.30W | 1874 - 2017 |
| Catamarca Aero | 8722200 | 28.60S | 65.77W | 1904 - 2017 |
| Gore | 8714900 | 26.82S | 60.45W | 1926 - 2014 |
| Asunción Aero | 8621800 | 25.25S | 57.52W | 1893 - 2017 |
| Las Lomitas | 8707800 | 24.70S | 60.58W | 1951 - 2017 |
| Rivadavia | 8706500 | 24.17S | 62.90W | 1931 - 2017 |
| Oran Aero | 8701600 | 23.15S | 64.32W | 1961 - 2017 |
| La Quiaca | 8700700 | 22.10S | 65.60W | 1911 - 2017 |
| Yacuiba | 8536500 | 21.95S | 63.65W | 1914 - 2017 |
| Mariscal Estig | 8606800 | 22.02S | 60.60W | 1951 - 2017 |
| Camiri | 8531500 | 20.00S | 63.53W | 1951 - 2017 |
| Potosi | 8529300 | 19.55S | 65.73W | 1943 - 2017 |
| *Precipitation* |  |  |  |  |
| Yuto | 8701607 | 23.38S | 64.28W | 1934 - 1990 |
| Caimancito | 8701621 | 23.73S | 64.47W | 1947 - 2007 |
| La Estrella | 8701608 | 23.82S | 64.08W | 1941 - 1990 |
| Calilegua | 8704616 | 23.78S | 64.77W | 1935 - 1989 |
| Ledesma | 8704618 | 23.83S | 64.78W | 1928 - 2002 |
| Fraile Pintado | 8704603 | 23.95S | 64.78W | 1935 - 1990 |
| La Esperanza | 8704628 | 24.22S | 64.85W | 1900 - 2002 |
| Gral. Pizarro | 8704605 | 24.22S | 64.02W | 1941 - 1990 |
| Jujuy Aero | 8704600 | 24.38S | 65.08W | 1931 - 2017 |
| Jujuy | 8704607 | 24.30S | 65.30W | 1908 - 2002 |
| Salta Aero | 8704700 | 24.85S | 65.48W | 1873 - 2017 |
| Urunde | 8701606 | 23.55S | 64.40W | 1934 - 1990 |
| Pichanal | 8701620 | 23.32S | 64.23W | 1934 - 1990 |
| Embarcación | 8701609 | 23.22S | 64.12W | 1928 - 2002 |
| Oran Aero | 8701600 | 23.15S | 64.32W | 1915 - 2017 |
| Aguas Blancas | 8701612 | 22.72S | 64.36W | 1945 - 2012 |
| Tartagal Aero | 8702200 | 22.65S | 63.82W | 1935 - 2017 |
| Yacuiba | 8536500 | 21.95S | 63.65W | 1914 - 2017 |
| La Quiaca | 8700700 | 22.10S | 65.60W | 1903 - 2017 |
| Tarija | 8536400 | 21.55S | 64.70W | 1942 - 2017 |
