# Supplementary material for: Hantavirus pulmonary syndrome outbreaks associated with climate variability in Northwestern Argentina, 1997–2017
Source: PLoS Negl Trop Dis. 2020 Nov 30;14(11):e0008786. doi: 10.1371/journal.pntd.0008786 (PMC7728390; doi:10.1371/journal.pntd.0008786)
Supplement: S3 Table — (DOCX) [file pntd.0008786.s003.docx]

| Biannual Models | AICc | ∆ AICCc | RMSE | R^2^_adj_ |
| --- | --- | --- | --- | --- |
| Rainfall(t-1), Temperature(t-1), AR 2 | 90.8 | 0 | 0.982 | 0.50 |
| Rainfall(t-1), Temperature(t-2) | 91.2 | 0.4 | 1.036 | 0.34 |
| Rainfall(t-2), Temperature(t-3), MA1 | 91.2 | 0.4 | 1.031 | 0.35 |
| Rainfall(t-1), Temperature(t-0) | 91.2 | 0.4 | 1.036 | 0.34 |
| Rainfall(t-1, t-2), Temperature(t-2) AR 2 | 93.3 | 2.5 | 0.898 | 0.51 |
| Rainfall(t-0), Temperature(t-1), AR 2 | 95.6 | 4.8 | 1.018 | 0.34 |
| Quarterly Models |  |  |  |  |
| Rainfall(t-1), Temperature(t-0, t-1), AR 4 MA1 | 153.1 | 0 | 0.6438 | 0.53 |
| Rainfall(t-3), Temperature(t-3), AR 4 M1 | 154.1 | 0.99 | 0.657 | 0.51 |
| Rainfall(t-1), Temperature(t-0, t-2), AR 1 | 155.3 | 2.19 | 0.564 | 0.41 |
| Rainfall(t-0, t-1), Temperature(t-0, t-1), AR 4 MA1 | 155.8 | 2.62 | 0.6429 | 0.53 |
| Rainfall(t-1, t-2), Temperature(t-0, t-2) | 156.6 | 3.45 | 0.729 | 0.40 |
| Rainfall(t-1, t-2, t-3), | 158.0 | 4.89 | 0.706 | 0.43 |
| Bimestrial Models |  |  |  |  |
| Rainfall ( t-2), Temperature(t-1, t-2) AR 4 MA1 | 292.8 | 0 | 0.896 | 0.58 |
| Rainfall(t-1, t-2), Temperature(t-1, t-2), AR 2 MA 2 | 293.2 | 0.44 | 0.91 | 0.55 |
| Rainfall(t-0, t-1, t-2), Temperature(t-0, t-1, t-2), AR 2 MA 1 | 296.4 | 3.62 | 0.907 | 0.56 |
| Rainfall(t-2), Temperature(t-1), AR1, MA4 | 298.1 | 5.33 | 0.933 | 0.54 |
| Rainfall(t-1), Temperature(t-1), AR2, MA1 | 298.4 | 5.6 | 0.96 | 0.51 |
| Temperature(t-1), AR2, MA1 | 298.6 | 5.8 | 0.96 | 0.51 |
| Temperature(t-2), AR2, MA1 | 299.4 | 6.6 | 0.957 | 0.52 |

S3 Table. Model comparison for different combinations of lagged rainfall, temperature and ARIMA error based on the Corrected Akaike Inforemation Criterion (AICc) for hantavirus infections without outliers: 3 observation of the 2006/2007 and 2 observation of the 2015 outbreaks.

Coefficients estimated for the best-fitting model of hantavirus infections and the two explanatory climatic variables in northwestern Argentina.

| Biannual Model |  |  |  |
| --- | --- | --- | --- |
| Rainfall(t-1), Temperature( t-1) | Estimated | Standard error | p-value |
| Rainfall(t-1) | 2.17 | 0.46 | >0.01 |
| Temperature (t-1) | 2.39 | 1.37 | >0.02 |
| AR 1 | 0.27 | 0.15 | >0.05 |
| AR 2 | -0.56 | 0.16 | >0.01 |
| Rainfall(t-1), Temperature( t-2) |  |  |  |
| Rainfall(t-1) | 2.33 | 0.66 | >0.01 |
| Temperature (t-2) | -1.94 | 1.21 | >0.05 |
| Rainfall(t-2), Temperature( t-3) |  |  |  |
| Temperature( t-3) | -4.20 | 1.10 | >0.01 |
| Rainfall(t-1), Temperature( t-0) |  |  |  |
| Rainfall(t-1) | 2.17 | 0.67 | >0.01 |
| Temperature( t-0) | 1.84 | 1.14 | >0.05 |
| Quarterly Model |  |  |  |
| Rainfall(t-1), Temperature(t-0, t-1) |  |  |  |
| Rainfall (t-1) | 0.83 | 0.07 | >0.01 |
| Temperature (t-0) | 0.52 | 0.05 | >0.01 |
| Temperature (t-1) | -0.38 | 0.06 | >0.01 |
| AR 1 | -0.54 | 0.12 | >0.01 |
| AR 2 | 0.33 | 0.11 | >0.02 |
| MA 4 | 0.89 | 0.09 | >0.01 |
| Rainfall(t-3), Temperature( t-3) |  |  |  |
| Rainfall(t-3) | 0.74 | 0.06 | >0.01 |
| Temperature( t-3) | -0.94 | 0.06 | >0.01 |
| AR 1 | -0.46 | 0.12 | >0.01 |
| AR 2 | 0.37 | 0.13 | >0.01 |
| AR4 | -0.43 | 0.11 | >0.01 |
| MA 4 | 0.91 | 0.10 | >0.01 |
| Bimestrial Models |  |  |  |
| Rainfall(t-2), Temperature(t-1, t-2) |  |  |  |
| Rainfall(t-2) | 0.47 | 0.16 | >0.01 |
| Temperature (t-1) | 0.53 | 0.11 | >0.01 |
| Temperature (t-2) | -0.51 | 0.18 | >0.01 |
| AR 1 | -0.38 | 0.11 | >0.01 |
| AR 2 | -0.61 | 0.11 | >0.01 |
| AR 4 | -0.26 | 0.11 | >0.05 |
| MA 1 | 0.88 | 0.06 | >0.01 |
| Rainfall(t-1, t-2), Temperature(t-1, t-2) |  |  |  |
| Rainfall(t-1) | 0.29 | 0.18 | >0.05 |
| Rainfall(t-2) | 0.49 | 0.16 | >0.01 |
| Temperature (t-1) | 0.36 | 0.14 | >0.05 |
| Temperature (t-2) | -0.65 | 0.19 | >0.01 |
| AR 1 | -0.41 | 0.09 | >0.01 |
| AR 2 | -0.42 | 0.10 | >0.01 |
| MA 1 | -0.91 | 0.06 | >0.01 |

Only significant coefficients are listed, all estimated for standardized z- values, in first seasonal difference. AR: Autoregressive and MA: Moving average component of the ARIMA error term component.
